# Comparing 5G mobile stroke unit and emergency medical service in patients acute ischemic stroke eligible for t‐PA treatment: A prospective, single‐center clinical trial in Ya'an, China

**DOI:** 10.1002/brb3.3231

**Published:** 2023-08-25

**Authors:** Bo Zheng, Yan Li, Gangfeng Gu, Jian Yang, Junyao Jiang, Zhao Chen, Yang Fan, Sheng Wang, Han Pei, Jian Wang

**Affiliations:** ^1^ Department of Neurology Ya'an Peoples Hospital Ya'an China

**Keywords:** acute ischemic stroke, emergency medical services, mobile stroke unit, prognosis, thrombolysis

## Abstract

**Background:**

This study aims to assess and compare the functional outcomes of patients with acute ischemic stroke (AIS) eligible for tissue plasminogen activator (t‐PA) treatment who received care from either a fifth‐generation(5G) mobile stroke unit (MSU) or traditional emergency medical service (EMS).

**Method:**

The study recruited patients between February 2020 and January 2022, with the final 90‐day follow‐up concluded in April 2022. Prior to enrollment, patients were assigned to either EMS or MSU care based on predetermined rules. The primary outcome measure was the Modified Rankin Scale (mRS) score at 90 days, with secondary outcome measures including time metrics, mRS and National Institutes of Health Stroke Scale scores at 7‐day follow‐up, and hospitalization costs.

**Results:**

Of the 2281 enrolled patients, 207 were eligible for t‐PA treatment, with 101 allocated to MSU care and 106 to EMS care. The percentage of patients achieving a favorable mRS score (0–2) at 90 days was 82.2% in the MSU group compared to 72.6% in the EMS group (*p <* .05). Median times from symptom onset to thrombolysis were 146 min in the MSU group and 204 min in the EMS group, while median times from ambulance alert to computed tomography (CT) completion were 53 and 128 min, respectively. Hospitalization charges averaged approximately $3592 in the MSU group and $4800 in the EMS group.

**Conclusions:**

Our findings indicate that 5G MSU care significantly reduces the time from symptom onset to stroke diagnosis and intravenous thrombolysis in patients with AIS, resulting in improved functional outcomes compared to EMS care. As China continues its deployment of 5G technology and other digital infrastructures, the adoption of 5G MSU care on a broader scale may eventually supplant traditional stroke treatment approaches.

## Graphical abstract

The prognosis of patients with acute ischemic stroke is related to the time of thrombolytic therapy. The use of fifth‐generation (5G) mobile stroke units can significantly shorten treatment time and improve patient prognosis. The purpose of this study is to investigate whether patients with acute ischemic stroke who are eligible for t‐PA treatment have better functional results after receiving 5G mobile stroke unit care than receiving emergency medical services.

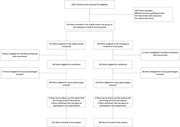

## 1 INTRODUCTION

According to the China Stroke Prevention and Treatment Report (2018) (Wang et al., 2022), the prevalence of stroke among Chinese residents aged 40 years and over experienced an increase from 1.89% to 2.19% between 2012 and 2016. As a leading cause of mortality and disability among middle‐aged and elderly individuals in China, stroke poses a significant global public health threat due to its high incidence, recurrence, mortality, and disability rates (Harper et al., 1992). Regrettably, once a stroke occurs, the damage incurred is irreversible (Saver, 2006). Intravenous thrombolysis has become the standard treatment for acute ischemic stroke (AIS) and should be administered within 6 h of symptom onset to prevent irreversible necrosis of brain tissue (Berge et al., 2021; Larsen et al., 2021; Turc et al., 2019). Timely treatment is crucial for promoting early recovery and improving patients’ quality of life post‐discharge (Gumbinger et al., 2014; Hacke et al., 2004; Tung et al., 2011).

In an effort to reduce the time from symptom onset to thrombolytic therapy, the world's first mobile stroke unit (MSU) was successfully developed by Saarland University Hospital (Humboldt, Germany) in 2010, revolutionizing traditional emergency medical service (EMS) models (Fassbender et al., 2017; Parker et al., 2015). Studies have demonstrated that MSUs can effectively reduce treatment delay, enabling more patients to receive reperfusion therapy within the critical “golden time” window (Ebinger et al., 2014; Fassbender et al., 2003; Hacke et al., 2008). Although the adoption of MSUs in China was relatively recent, with the first introduction in August 2017 by Henan Provincial People's Hospital, they have gained increasing popularity in various Chinese cities, such as Zhangjiakou, Liaocheng, Liuyang, Ya'an, Ulanhot, and others (Xu & Zhao, 2018). Furthermore, with the integration of fifth‐generation mobile communication technology (5G) in the medical field, 5G MSUs have emerged, combining the advantages of 5G technology with the capabilities of MSUs to enhance system configuration and communication. This integration enables hospital expert groups to provide real‐time video guidance for patient treatment during ambulance transportation (Audebert et al., 2017; Handschu et al., 2003; Neural Injury & Repair Branch of Chinese Neuroscience Society et al., 2019).

Based on the regional geographical characteristics of Ya'an, China, the research team at Ya'an People's Hospital has developed a multifunctional 5G smart mobile stroke treatment platform. The objective of this study is to investigate whether AIS patients eligible for 5G MSU care achieve superior functional outcomes compared to those receiving EMS care.

## 2 METHODS

The research protocol (202024) for this study was developed in compliance with the revised Helsinki Declaration on Human Biomedical Research, Good Clinical Practice Guidelines, and local laws and regulations. The protocol was evaluated and approved by the Ethics Committee of Ya'an People's Hospital on February 12, 2020. Prior to inclusion in the study, informed consent was obtained from all recruited patients after thoroughly explaining the study protocol to them. The investigators responsible for outcome evaluation, data management, and analysis were blinded to patient enrollment and treatment processes. The study was registered with the Chinese Clinical Trial Registry (ChiCTR) on November 5, 2020 (ChiCTR2000039695). Further details regarding the filtering and registration process can be found in the study protocol.

### 2.1 Study design

**FIGURE 1:**
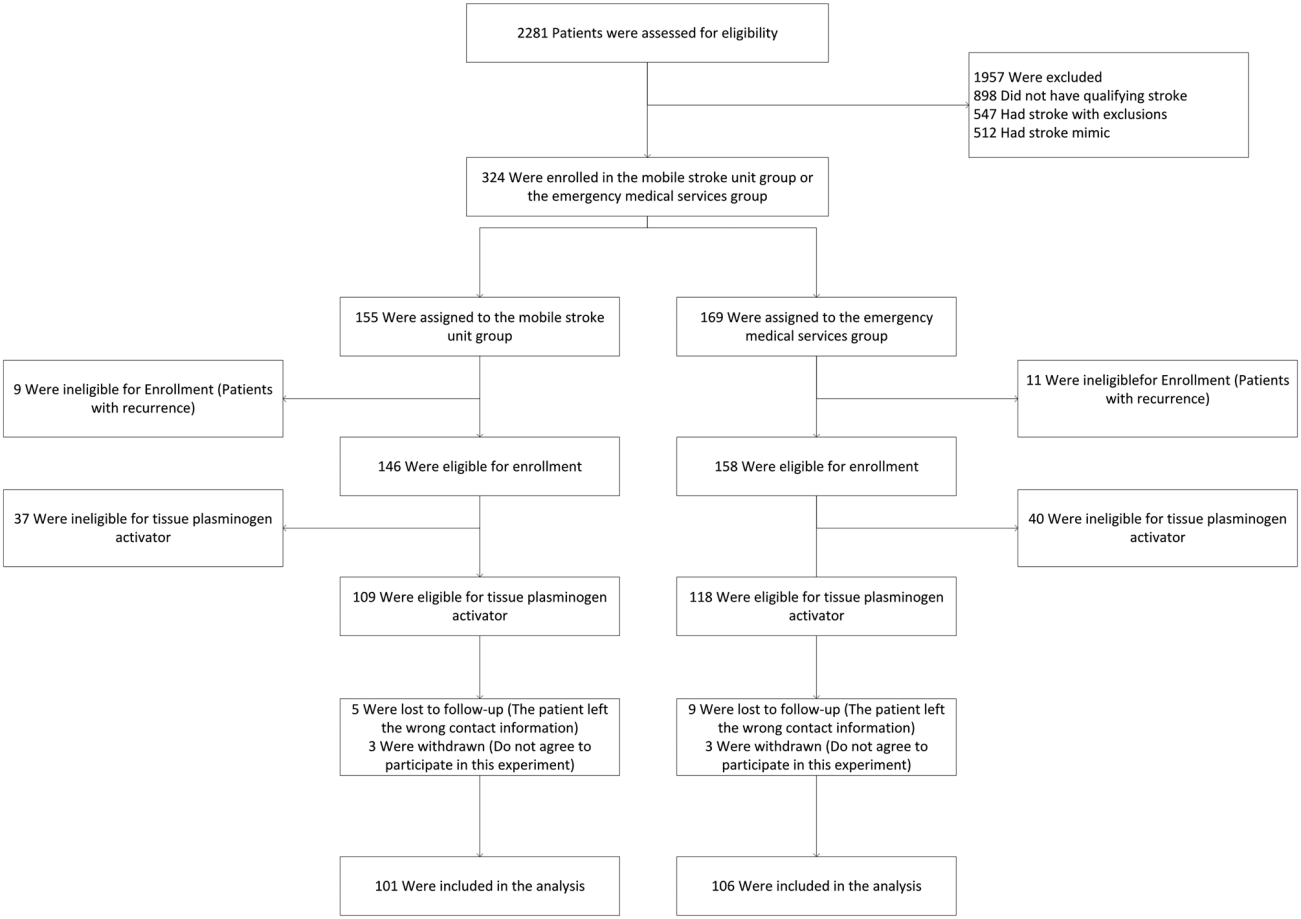
Flow chart of the experiment.

This study employed a prospective design to compare the outcomes of patients with AIS who received 5G MSU care with those who did not. The 5G MSU is equipped with non‐spiral computerized tomography (CT) and related diagnostic instruments, serving as a prehospital emergency system that integrates neurological examination, CT diagnosis, and intravenous thrombolytic therapy. Prior to enrollment, patients were assigned to receive either EMS or 5G MSU care. Ambulance services were provided by Ya'an People's Hospital. For practical reasons, a two‐shift schedule was implemented for both 5G MSU and EMS, with day shifts from 8 a.m. to 8 p.m. and night shifts from 8 p.m. to 8 a.m. The 5G MSU and EMS shifts alternated weekly. Follow‐up assessments of patients were scheduled at day 7 and day 90 post‐discharge, with follow‐up data collected through the WeChat app on their phones. Group assignment was not blinded for data collection. Participants were excluded if they were ineligible for tissue plasminogen activator (t‐PA) treatment or if they were not accessible for follow‐up data (Figure 1).

### 2.2 Trial population

**FIGURE 2:**
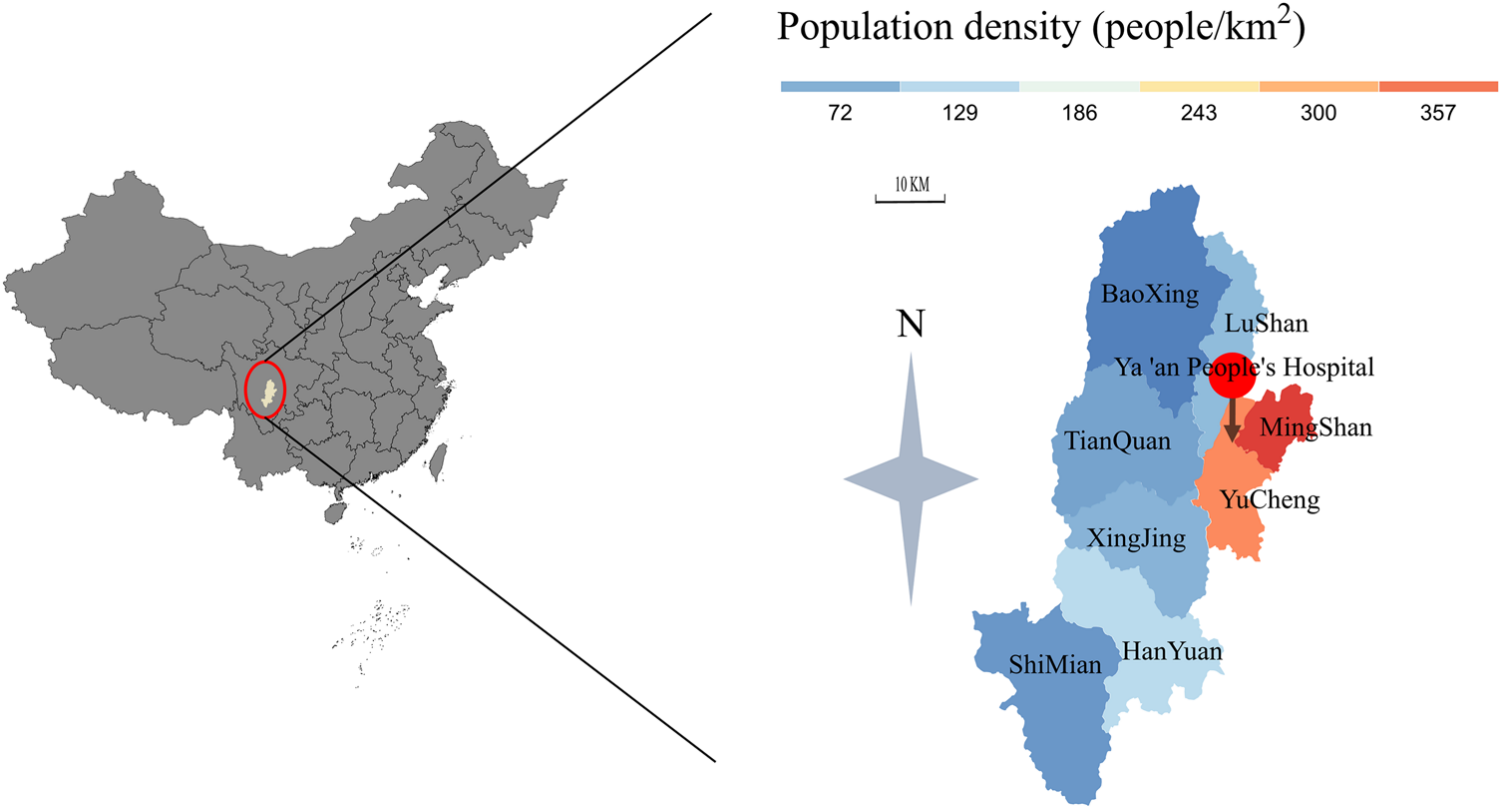
Geography map based on population density of Ya'an.

The study was conducted in Ya'an, a city located in southwestern China, spanning approximately 15,000 km^2^ and with a population exceeding 1.6 million (Figure 2). Patients with AIS who met the inclusion criteria were recruited, which included being aged 18 years or older, presenting within 6 h from stroke onset, having a final diagnosis of transient ischemic attack or AIS, and providing written informed consent. Exclusion criteria encompassed patients whose symptoms resolved upon ambulance (or 5G MSU) arrival, those with malignancy or severe primary diseases, pregnant or lactating women, individuals with missing follow‐up data, and patients deemed unsuitable for trial participation due to other reasons. Written informed consent was obtained from all patients prior to their inclusion in the study, covering prehospital thrombolysis, follow‐up, and inclusion in the registry.

### 2.3 Interventions

**FIGURE 3:**
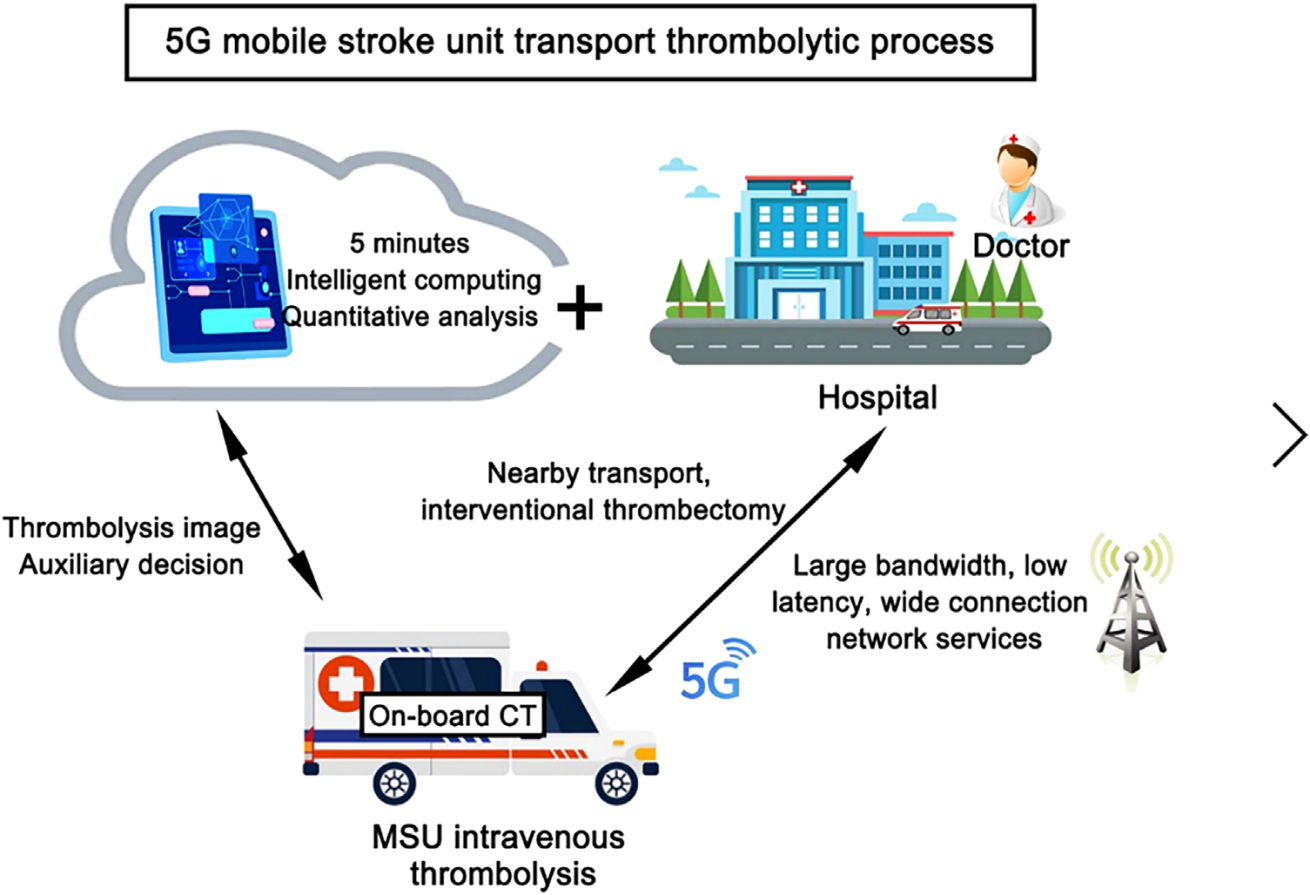
Fifth‐generation (5G) mobile stroke unit transport thrombolytic process.

The 5G MSU was staffed with an emergency physician, a radiologist, and a paramedic. Both the emergency physician and the radiologist underwent 3 years of professional training and passed relevant examinations. EMS was staffed with a paramedic and an emergency physician, both of whom received professional training. Prehospital diagnostics for patients in the MSU group commenced either on‐site or within the 5G MSU ambulance. Upon arriving at the emergency scene, the medical personnel in the 5G MSU conducted immediate tests to confirm the stroke condition and engaged in real‐time remote consultations with neurologists to formulate appropriate rescue strategies (Figure 3). Patients assigned to the EMS group were transported to an ambulance for routine medical evaluation and basic first aid measures. If a suspected acute stroke patient was admitted to the hospital, a CT scan was performed to confirm eligibility for thrombolytic treatment.

### 2.4 Outcomes

The primary outcome measure of this study was the Modified Rankin Scale (mRS) score at the 90‐day follow‐up, assessed through structured interviews incorporating highly consistent and reliable clinical observations. It was hypothesized that patients receiving 5G MSU care would exhibit lower mRS scores compared to those receiving EMS care. Secondary outcomes included the mRS score at 7 days, the National Institutes of Health Stroke Scale (NIHSS) score at 7 days, time duration from ambulance alert to hospital arrival, time duration from ambulance alert to completion of CT imaging, time duration from symptom onset to t‐PA treatment initiation, time duration from CT imaging to t‐PA treatment initiation, duration of t‐PA treatment, and hospitalization costs.

### 2.5 Statistical analysis

Intention‐to‐treat analyses were performed using IBM SPSS Version 27, with a statistical significance level set at .05. Data analysis employed chi‐square tests, Student's *t*‐tests, Fisher's exact tests, and Wilcoxon rank‐sum tests. The mRS scores were analyzed as an ordinal scale (0–6) and dichotomized, with mRS scores of 0–2 indicating a favorable outcome (functionally independent), scores of 3–5 representing a poor prognosis, and a score of 6 indicating death. Adjusted and unadjusted binary logistic regressions were employed to analyze the outcomes, with predictors selected through univariate analyses and clinical evaluations. Cases in which participants in the experimental group dropped out before the end of the study were considered failures, and participants who violated the protocol during intervention or follow‐up were excluded from the analysis. Multiple estimations were conducted to address missing data, utilizing baseline variables as auxiliary variables.

## 3 RESULTS

### 3.1 Descriptive findings

The study was conducted from February 2020 to January 2023, with a total of 2281 patients screened. Ultimately, 207 AIS patients eligible for t‐PA treatment were enrolled, comprising 101 patients (48.8%) in the MSU group and 106 patients (51.2%) in the EMS group (Figure 1). Baseline characteristics, except for diabetes, were similar between the MSU and EMS groups, including stroke severity, aphasia, and left/right side weakness (Table 1). In the MSU group, the mean age of patients was 70.1 ± 11.7 years, with 46% being females. The EMS group had a mean age of 70.7 ± 10.3 years, with 35% being females. Medical history did not significantly differ between the groups, except for diabetes. The distribution of NIHSS scores exhibited similar trends between the two groups. The percentage of patients receiving treatment within 4.5 h after symptom onset was 97.0% in the MSU group and 94.3% in the EMS group, without statistical significance (*p* > .05).

**TABLE 1:** Baseline Parameters and clinical information in patients.

Characteristic	MSU group (*n* = 101)	EMS group (*n* = 106)	*p*‐Value
Female, *n* (%)	46 (45.5)	37 (34.9)	.317
Mean age (SD) (years)	70.1 ± 11.7	70.7 ± 10.3	.975
Median weight (SD) (kg)	60.4 ± 10.3	62.3 ± 9.8	.138
Medical history/risk factors, *n* (%)			
Hypertension	60 (59.4)	60 (56.6)	.791
Atrial fibrillation	24 (23.8)	21 (19.8)	.505
Diabetes	13 (12.9)	5 (4.7)	.048
Heart disease	11 (10.9)	11 (10.4)	1.000
Smoking	28 (27.7)	28 (26.4)	.876
Alcohol consumption	22 (21.8)	18 (17.0)	.482
TIA	13 (12.9)	5 (4.7)	.048
Large vessel occlusions	29 (26.4)	23 (21.7)	.265
Aphasia	41(40.6)	48 (45.3)	.274
Left/right side weakness	42 (41.6)/29 (28.7)	45 (42.5)/36 (34.0)	.551
First assessed NIHSS score			
Distribution, *n* (%)			
Median (IQR)	9 (4–17)	10.5 (7–16)	.333
0–5	33 (32.7)	24(22.8)	
6−14	37 (36.6)	53(50.1)	
≥15	31 (30.7)	29(27.1)	
Time of symptom onset			.499
<4.5h	98 (97.0)	100 (94.3)	
4.5–6 h	3 (3.0)	6 (5.7)	

Abbreviations: EMS, emergency medical service; IQR, interquartile range; MSU, mobile stroke unit; NIHSS, National Institutes of Health Stroke Scale; SD, standard deviation; TIA, transient ischemic attacks.

### 3.2 Primary outcome

**FIGURE 4:**
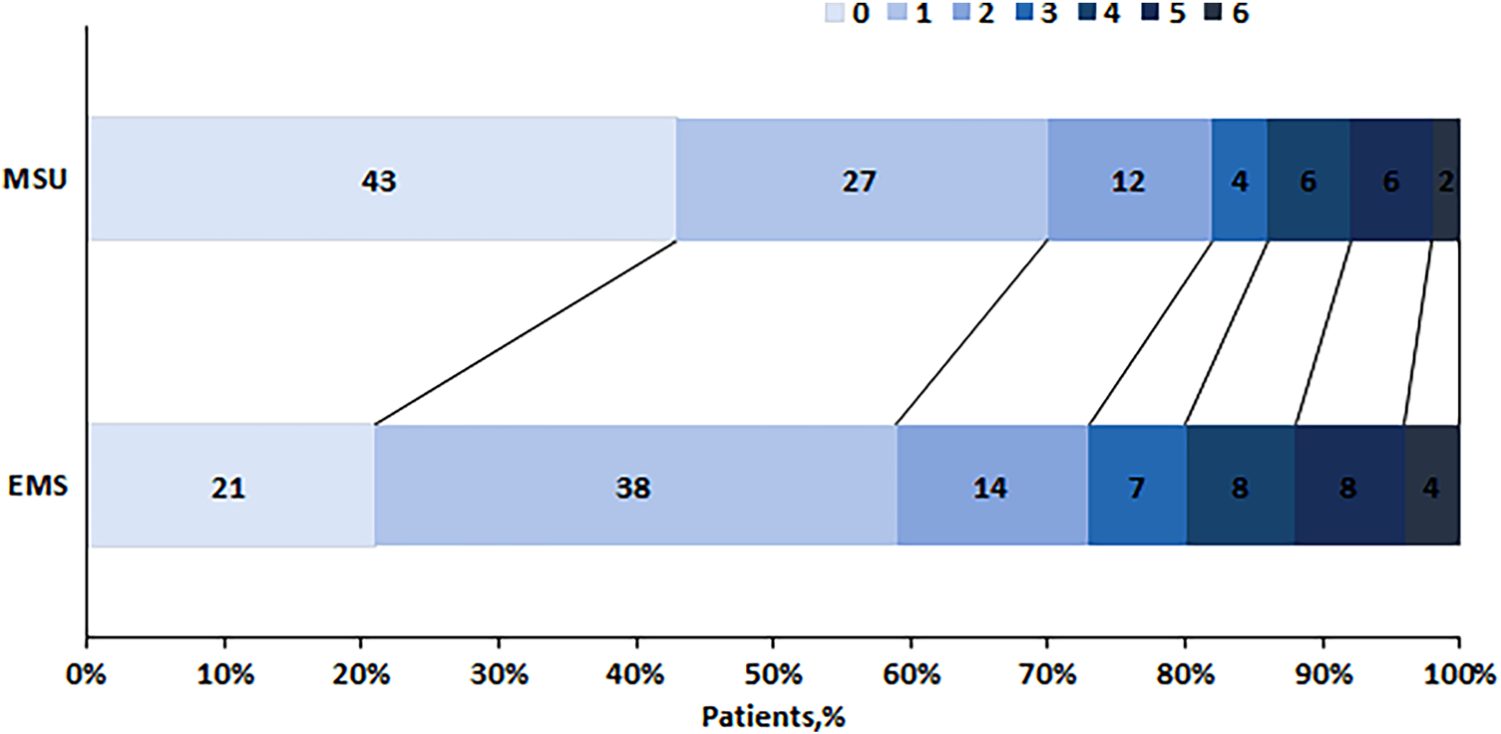
Primary outcome.

The primary outcome measure was the mRS score at 90 days for t‐PA‐eligible patients (Figure 4). The percentage of patients achieving an mRS score of 0–2 was 82.2% in the MSU group and 72.6% in the EMS group (*p <* .05). The percentage of patients with an mRS score of 3–5 was 15.8% in the MSU group and 23.6% in the EMS group. The percentage of patients with an mRS score of 6 was 2% in the MSU group and 3.8% in the EMS group (Table 2).

**TABLE 2:** Study outcomes.

	MSU group (*n* = 101)	EMS group (*n* = 106)	*p*‐Value
Primary outcome			
90‐Day mRS score			
Median (IQR)	1 (0–2)	1 (0–3)	.019
Distribution *n* (%)			
0–2	83 (82.2)	77 (72.6)	.007
3–5	16 (15.8)	25 (23.6)	.883
6	2 (2.0)	4 (3.8)	.498
Secondary outcomes			
7‐Day mRS score			
Median (IQR)	2 (0–4)	2 (1–5)	.028
Distribution *n* (%)			
0–2	66 (65.3)	58 (54.7)	
3–5	35 (34.7)	46 (43.4)	
6	0 (0)	2 (1.9)	
7‐Day NIHSS score			
Median (IQR)	2 (0–10)	3 (2–12)	.034
Distribution, *n* (%)			
0–5	62 (61.4)	57 (53.8)	
6−14	22 (21.8)	28 (26.4)	
≥15	17 (16.8)	19 (17.9)	
Hospitalization cost (US$)			
Mean (±SD)	3592 (±2920)	4800 (±5274)	.150

*Note*: $1 = 6.892 RMB.

Abbreviations: EMS, emergency medical service; IQR, interquartile range; MSU, mobile stroke unit; NIHSS, National Institutes of Health Stroke Scale.

### 3.3 Secondary outcomes

At 7 days, the median mRS score for patients was 2 (IQR: 0–4) in the MSU group and 2 (IQR: 1–5) in the EMS group (*p <* .05). The percentage of patients with an mRS score of 0–2 at 7 days was 65.3% in the MSU group and 54.7% in the EMS group, while the percentage of patients with an mRS score of 3–5 at 7 days was 34.7% in the MSU group and 43.4% in the EMS group. The median NIHSS score at 7 days was 2 (IQR: 0–10) in the MSU group and 3 (IQR: 2–12) in the EMS group (*p <* .05). Complete results are presented in Table 2. The average hospitalization cost was $3592 in the MSU group and $4800 in the EMS group (*p* > .05).

Table 3 displays the comparison of time metrics between t‐PA‐eligible patients in the MSU and EMS groups. Compared to the EMS group, the time duration from ambulance alert to completing CT imaging, from symptom onset to receiving t‐PA treatment, and from CT imaging to t‐PA treatment were all shorter in the MSU group, with all differences being statistically significant (*p <* .01).

**TABLE 3:** Time metrics in patients eligible for tissue plasminogen activator (t‐PA).

Interval	MSU (min)	EMS (min)	*p*‐Value
Median time from ambulance alert to arrive the hospital (IQR)	90 (49–117)	99 (48–148)	.179
Median time from ambulance alert to CT imaging (IQR)	53 (35–61)	128 (71.5–170.5)	.000
Median time from symptom onset to t‐PA treatment (IQR)	146.2 (126–192)	204.5 (138–280.75)	.000
Median time from CT imaging to t‐PA treatment (IQR)	4.75 (2–6)	21.5 (9.25–34)	.000
Median time of t‐PA treatment (IQR)	60 (58–63)	60 (40.25–61)	.006

Abbreviations: CT, computed tomography; EMS, emergency medical service; IQR, interquartile range; MSU, mobile stroke unit.

## 4 DISCUSSION

This study presents the findings of a prospective trial that aimed to compare the outcomes of patients diagnosed with AIS receiving care either from a 5G MSU or an EMS. To ensure comparability, the study ensured that the baseline characteristics of patients in both groups were similar, with the exception of diabetes. Data were collected over a 90‐day follow‐up period. The results demonstrated that patients in the MSU group exhibited lower mRS scores at 90 days, as well as at 7 days for both mRS and NIHSS scores. These findings suggest a more favorable prognostic outcome for patients in the MSU group compared to the EMS group. Additionally, the median time from symptom onset to thrombolysis was shorter in the MSU group, indicating a prompter administration of treatment. Furthermore, the study observed reduced hospitalization costs associated with MSU care.

The findings of our study are in line with previous research in the field. Ebinger et al. (2015) conducted a collaborative effort with the fire department to design a stroke emergency mobile unit (STEMO), and their study demonstrated that implementing STEMO increased the proportion of patients receiving thrombolytic therapy within the critical “golden hour,” leading to improved short‐term outcomes without compromising patient safety. Similarly, Grotta et al. (2021) reported higher rates of thrombolysis and lower rates of disability in patients treated with an MSU compared to those treated with EMS. The superior outcomes observed in the MSU group compared to the EMS group can potentially be attributed to the MSU's ability to enhance thrombolytic rates and reduce treatment duration (Ebinger et al., 2021). In the case of patients with AIS, timely intervention is crucial. Prior studies have demonstrated the benefits of early intravenous thrombolytic therapy following AIS onset in terms of facilitating patient rehabilitation (Lees et al., 2010; Powers, 2020; Saver et al., 2016). Moreover, the use of MSU has been associated with improved functional outcomes and reduced time from symptom onset to t‐PA treatment initiation (Turc et al., 2022). Furthermore, another study highlighted the long‐term medical benefits and cost‐effectiveness of MSU over EMS (Chen et al., 2022).

Although the average hospitalization cost in the MSU group was lower compared to the EMS group, the observed differences did not reach statistical significance. However, it is important not to overlook the efficacy of MSU in stroke management. Previous studies have demonstrated that the utilization of MSUs leads to improved quality‐adjusted life years for patients in comparison to EMS, indicating its potential as a cost‐effective option for patient care (Kim et al., 2021; Lund et al., 2022; Walter et al., 2018). Therefore, it is crucial to approach cost‐effectiveness analysis with caution, considering various factors that may influence the cost of implementing a 5G MSU system, such as clinical geographical factors, ambulance resource allocation, ambulance equipment costs, and training expenses for ambulance and emergency personnel (Ehlers et al., 2007; Reimer et al., 2020). Conducting a comprehensive cost‐benefit analysis is warranted to determine the clinical and operational value of MSUs in the context of stroke management.

One of the notable strengths of this study lies in the integration of 5G technology with MSUs, which opens up new possibilities for delivering emergency treatment to patients with stroke. However, several challenges remain that need to be effectively addressed during practical implementation. First, there is a need for further improvement in 5G technology infrastructure construction and the development of mobile stroke treatment teams. This includes ensuring the stability and safety of 5G transmission technology, as well as aligning the operation mode and qualification of 5G MSUs with existing local EMS. Second, additional research efforts are required to mitigate the misdiagnosis rates associated with 5G technology in stroke emergencies. Moreover, addressing staffing issues within MSUs due to the shortage of specialists in cerebrovascular diseases is crucial. Finally, exploring novel models for utilizing 5G MSUs, such as performing surgical procedures through 5G technology within the MSUs, and effectively integrating 5G MSUs into overall medical emergency management are avenues worth exploring.

Several limitations should be acknowledged in this study. First, the absence of randomization in the trial design may have led to biased treatment assignments, potentially impacting the internal validity of the findings. Second, the evaluation of patients through the WeChat app on the phone introduced limitations in data accuracy compared to face‐to‐face communication. However, efforts were made to mitigate this risk by maintaining standardized records. Additionally, although the number of missing follow‐up data was small, there remains a certain risk of bias associated with these missing data. Lastly, the single‐center nature of the trial and the relatively small sample size restrict the generalizability and external validity of the results, warranting caution when interpreting the findings in a broader context.

## 5 CONCLUSSION

This study emphasizes the substantial advantages of 5G MSU management in reducing waiting times for patients with AIS, enabling timely stroke diagnosis and intravenous thrombolysis, thereby contributing to improved functional prognosis compared to EMS. With the ongoing deployment of 5G technology and the increasing prevalence of information and communication technologies, such as artificial intelligence and edge computing, there is a growing potential for the digitalization, intelligence, and collaboration of MSUs and in‐hospital stroke centers. This transformative landscape presents promising opportunities for the development of 5G MSUs, which hold significant promise for eventually replacing conventional stroke treatment modalities on a larger scale.

## AUTHOR CONTRIBUTIONS

Bo Zheng and Jian Wang designed the study. Yan Li and Yang Fan conducted statistical analyses. Gangfeng Gu, Yan Li, Jian Yang, Junyao Jiang, Zhao Chen, Yang Fan and Sheng Wang contributed to data collection. Bo Zheng and Yan Li wrote the paper. Bo Zheng, Han Pei and Jian Wang revised the manuscript. All authors approved the final draft.

## CONFLICT OF INTEREST STATEMENT

The authors declare no conflict of interest.

## CLINICAL TRIAL REGISTRATION

This study was registered at Chinese Clinical Trial Registry (ChiCTR) with registration number: ChiCTR2000039695.

### PEER REVIEW

The peer review history for this article is available at https://publons.com/publon/10.1002/brb3.3231.

## Data Availability

The data that support the findings of this study are available on request from the corresponding author. The data are not publicly available due to privacy or ethical restrictions.
